# Validation of the Prevention Impacts Simulation Model (PRISM)

**DOI:** 10.5888/pcd18.200225

**Published:** 2021-02-04

**Authors:** Benjamin Yarnoff, Amanda Honeycutt, Christina Bradley, Olga Khavjou, Laurel Bates, Sarah Bass, Rachel Kaufmann, Lawrence Barker, Peter Briss

**Affiliations:** 1 RTI International, Research Triangle Park, North Carolina; 2 Centers for Disease Control and Prevention, Atlanta, Georgia

## Abstract

**Introduction:**

Demonstrating the validity of a public health simulation model helps to establish confidence in the accuracy and usefulness of a model’s results. In this study we evaluated the validity of the Prevention Impacts Simulation Model (PRISM), a system dynamics model that simulates health, mortality, and economic outcomes for the US population. PRISM primarily simulates outcomes related to cardiovascular disease but also includes outcomes related to other chronic diseases that share risk factors. PRISM is openly available through a web application.

**Methods:**

We applied the model validation framework developed independently by the International Society of Pharmacoeconomics and Outcomes Research and the Society for Medical Decision Making modeling task force to validate PRISM. This framework included model review by external experts and quantitative data comparison by the study team.

**Results:**

External expert review determined that PRISM is based on up-to-date science. One-way sensitivity analysis showed that no parameter affected results by more than 5%. Comparison with other published models, such as ModelHealth, showed that PRISM produces lower estimates of effects and cost savings. Comparison with surveillance data showed that projected model trends in risk factors and outcomes align closely with secular trends. Four measures did not align with surveillance data, and those were recalibrated.

**Conclusion:**

PRISM is a useful tool to simulate the potential effects and costs of public health interventions. Results of this validation should help assure health policy leaders that PRISM can help support community health program planning and evaluation efforts.

**Summary**

**What is known on this topic?**

The Prevention Impacts Simulation Model (PRISM), a system dynamics model that simulates health, mortality, and economic outcomes for the US population, has been used to support community-level strategic planning in several US communities and to evaluate the potential long-term effects of community initiatives to reduce chronic disease and its risk factors.

**What is added by this report?**

Demonstrating the validity of a public health simulation model helps to establish confidence in the accuracy and usefulness of a model’s results. Our evaluation of the validity of PRISM indicates that it adequately simulates the potential effects and costs of public health interventions.

**What are the implications for public health practice?**

Results should assure healthy policy leaders that PRISM can support community health program planning and evaluation efforts.

## Introduction

Public health approaches to address the growing prevalence of chronic conditions range from individual-level disease management interventions (eg, clinical pharmacists) to community interventions that target population subgroups (eg, smoking bans in workplaces) or that target whole populations (eg, initiatives to promote fruit and vegetable consumption). Decision makers in communities, local public health agencies, and other settings can benefit from tools to support planning for chronic disease prevention programs and evaluation of the potential long-term impact of implemented interventions. Although short-term evaluations of public health interventions are useful for assessing what interventions were implemented, how many people were reached, and short-term changes in health behaviors or outcomes, a longer analysis time frame is needed to evaluate or project long-term changes in chronic disease outcomes.

Simulation models can be useful in assessing the potential impact and cost-effectiveness of public health interventions over periods longer than the typical 1- to 5-year funding periods (10 years or more). Model projections can also inform planning decisions about which interventions to implement and how much funding to allocate to each intervention to achieve public health goals with limited resources. However, the usefulness of models for evaluation and policy planning rests partly on whether a potential user has confidence and trust in a model’s predictions (1). The primary method for ensuring confidence in a model is through model validation, which involves applying a set of approaches to assess how well a model predicts the health policy outcomes of interest to a decision maker (1).

The purpose of this study was to validate the current version of the Prevention Impacts Simulation Model (PRISM). PRISM was originally developed in 2005 to analyze the potential impacts of strategies to address cardiovascular disease (CVD) risk factors. PRISM is a population-level, mathematical model that synthesizes effect estimates from the literature and prevalence estimates from surveillance data sources such as the National Health and Nutrition Examination Survey (NHANES) to simulate health, mortality, and economic outcomes for the United States as a whole and for 6 community profiles defined by demographics related to population size, age group, and race and ethnicity. In this validation we focused on the nationally representative model.

**Figure 1:**
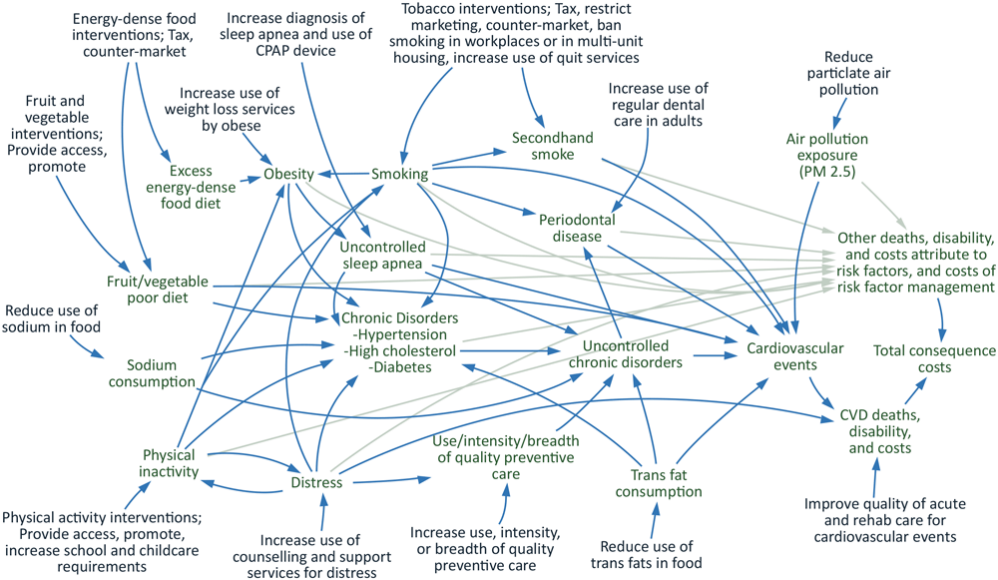
Diagram of the PRISM model. Abbreviations: CPAP, continuous positive airway pressure; CVD, cardiovascular disease; PRISM, Prevention Impacts Simulation Model.

PRISM is a system dynamics model that simulates the flow into and out of populations with chronic conditions and risk factors. For example, the population with diabetes changes based on inflows of incident diabetes cases and outflows of people with diabetes who die. Figure 1 provides an overview of the model structure including the relationship between intervention strategies, risk factors, and outcomes. PRISM primarily simulates outcomes related to CVD, but it also includes outcomes related to other chronic diseases that share risk factors, such as some cancers. The simulations are run annually from 2010 through 2060. PRISM produces estimates of the effect of implementing chronic disease prevention or management strategies compared with the status quo. Examples of strategies that can be examined using PRISM include workplace smoking bans, efforts to increase access to high-quality nutrition and physical activity, and evidence-based programs to manage diabetes, hyperlipidemia, and hypertension. (The PRISM technical guide is available in the resources section at https://prism-simulation.cdc.gov/app/cdc/prism/#/). PRISM has been used to support community-level strategic planning in several US communities (2–4) and to evaluate the potential long-term effects of community initiatives to reduce chronic disease and its risk factors (3,5,6).

Over the years, many disease prevention strategies and health and economic outcomes have been added to support the application of PRISM for policy planning and evaluation. For example, in 2016 a strategy to ban smoking in multi-unit housing was added to ensure that this priority strategy was among the options available to groups using PRISM for strategic planning and evaluation. Before 2020, PRISM was only available as part of Centers for Disease Control and Prevention (CDC) efforts to support chronic disease prevention program planning and evaluation for selected programs. However, the current version of PRISM, v3s3, is publicly available (https://prism-simulation.cdc.gov/app/cdc/prism/#/) via CDC funding. As a result, additional community health planning groups can now use PRISM to inform decisions about chronic disease prevention and management strategies for their communities. Evidence of the validity of PRISM can increase the confidence of potential users that results of the model simulation can be usefully applied to inform policy decisions.

## Methods

We used the recommended framework for model validation created by the International Society for Pharmoeconomics and Outcomes Research and the Society for Medical Decision Making for our analysis (1). This framework includes 5 types of validation for assessing a model: face validation, internal validation, cross validation, external validation, and predictive validation (1).

Face validation is the assessment by outside experts of a model’s structure, data sources, formulas, and results. Internal validation involves the verification of whether mathematical calculations in a model are implemented correctly by examining model code, having independent programmers write and compare code, conducting sensitivity analyses, and ensuring that programming code is efficient. Cross validation is the comparison of model output to output from other models. External validation is the comparison of model output to surveillance data on the same measures. Predictive validation is the comparison of predicted impacts from a model to real-world observations of the impact of an intervention (1).

In 2019, we conducted validity checks of PRISM version 3q1a, focusing on face, internal, cross, and external validation. Predictive validation was not included because of the lack of real-world long-term (ie, 10 years or more) follow-up data on the impact of the health policies and interventions simulated in PRISM, which limits confidence in model results for prediction. However, we have sought to fill this gap by using out-of-sample surveillance data in external validation.

### Face validation

To conduct face validation of PRISM version 3q1a, CDC subject matter experts (SMEs) on heart disease, diabetes, smoking, and nutrition, physical activity, and obesity reviewed PRISM model structure, equations, input values, and data sources for inputs that varied most widely in the 1-way sensitivity analysis conducted as part of internal validation. 

The study team provided each SME with a review package that included PRISM model documentation relevant to their area of expertise. Because PRISM includes more than 3,000 inputs, SMEs reviewed only those that had the greatest impact on key outcomes in 1-way sensitivity analyses (described in the internal validation section). To provide SMEs with comprehensive technical materials on PRISM, we added the equations underlying PRISM’s causal structure to the model technical documentation. We provided detailed instructions on what to review and a questionnaire for reporting comments on model structure and model parameters that should be updated. In addition, a second round of CDC SMEs reviewed all PRISM documentation to assess its scientific validity as part of CDC’s clearance process for making the tool publicly available. We updated model parameters based on SME suggestions from both rounds of review.

### Internal validation

For internal validation, we had a secondary programmer review all model code and resolve all questions with the primary programmer. We also conducted 1-way sensitivity analyses to examine the impact on deaths, cardiovascular events, and medical costs of using the highest and lowest plausible values for each PRISM parameter (determined from the literature and SMEs) compared with the default parameter value. We used 1-way sensitivity analyses to examine how assumptions about specific parameters underlying modeled relationships drive results for all strategies modeled in PRISM. (A full list of the parameters examined in the sensitivity analysis is available upon request.) For this exercise, we moved all PRISM strategy levers to their maximum, which resulted in more than 15 million premature deaths averted from 2018 to 2040. We also examined sensitivity of individual strategy levers and results were similar (not reported). In sensitivity analysis, if the estimated impact of strategies does not change substantially based on the range of any specific parameters, then it demonstrates that variation in that parameter is not a concern for model results. One-way analyses assume that all inputs except the one under consideration remain at their default values.

### Cross validation

In 2016, we conducted cross validation of the then-current version of PRISM (version 3q) by comparing simulated cardiovascular events and deaths with comparable results from 2 other simulation models for CVD: the CVD Policy Model (eg, Bibbins-Domingo et al [7]) and ModelHealth: CVD Microsimulation Model (eg, Dehmer et al [8]). This effort was part of a CDC study to explore the potential 5- and 10-year impacts of achieving Million Hearts (9) goals for aspirin use, blood pressure control, cholesterol control, sodium reduction, and smoking cessation and prevention (10). Results for cardiovascular events were similar across models. PRISM produced estimates for costs that were more conservative than the other 2 models.

Because the only change in PRISM between version 3q and version 3q1a was to add a new strategy lever for smoke-free multi-unit housing, the 2016 cross validation findings for clinical interventions are still applicable. To further cross validate PRISM results against other models, we searched the literature to identify models that analyzed other interventions included in PRISM version 3q1a. We sought models that focused on physical activity, nutrition, or smoking interventions and identified 3 models with published results for comparison with PRISM findings: a tobacco intervention microsimulation model (11), the Coronary Heart Disease Policy Model, which was used to analyze the impact of a tax on sugar-sweetened beverages (12), and the Childhood Obesity Intervention Cost-Effectiveness Study (CHOICES) microsimulation model, which has been used to analyze childhood physical activity (13). Maciosek et al (11) used a simulation model to estimate the potential long-term benefits of an intervention providing smoking cessation counseling to a cohort of 4 million adults. We used PRISM to simulate comparable results by estimating the impact of universal smoking cessation counseling on US adult smokers over a 30-year period. Wang et al (12) used the Coronary Heart Disease Policy Model to estimate the potential effects for adults aged 25 to 64 years of a national penny-per-ounce tax on sugar-sweetened beverages over a 10-year period. We used PRISM to simulate a comparable national tax on sugar-sweetened beverages using PRISM’s calorie-dense food tax lever, estimated as the tax scaled by the fraction of junk food consumption made up by sugar-sweetened beverages. Gortmaker et al (13) used the CHOICES model to estimate the potential long-term benefits of 2 childhood physical activity interventions over 10 years: a state policy requiring all public elementary schools to devote 50% of physical education time to moderate and vigorous physical activity and a state policy requiring all early childhood education centers to increase physical activity. We used PRISM to simulate a comparable physical activity intervention using PRISM’s physical activity in schools and physical activity in childcare levers.

### External validation

We compared PRISM results with recent data from national surveys and surveillance systems. Previous validations analyzed PRISM version 3q output compared with national estimates for 1990 through 2010. Because more recent data became available for comparison with PRISM output, we extended the period for external validation through 2016 and assessed how well PRISM version 3q1a output matched surveillance data.

Table 1 shows the PRISM output measures included in external validation, comparable national data sources, and time periods included. (Detailed methods for how each measure was calculated are available upon request.) For each measure, we graphed the simulated PRISM outcomes and the corresponding surveillance data or weighted estimates from national surveys. Because PRISM analyzes adults with a prior CVD event (ie, post-CVD) separately from those with no prior CVD event (ie, non-CVD), we analyzed outputs separately for these subpopulations whenever possible. If trends in output measures in PRISM deviated substantially from surveillance data, we recalibrated the model to better track with surveillance data. We recalibrated only in the case of substantial deviations to avoid over-calibrating the model.

**Table 1:** External Validation of PRISM Outputs and Comparable Measures Analyzed in Surveillance Data

Measure	Data Source and Years
Youth obesity (aged 2–17 y), %	NHANES 2011–2012, 2013–2014, 2015–2016; Skinner and Skelton (16)
Adult obesity, %	NHANES 2011–2012, 2013–2014, 2015–2016
Current smoker, %	
Former smoker, %	
Secondhand smoke exposure among nonsmoking adults, %	NHANES 2005–2006, 2007–2008, 2009–2010, 2011–2012, 2013–2014, 2015–2016
High blood pressure, %	NHANES 2011–2012, 2013–2014, 2015–2016
Borderline high blood pressure, %	
High cholesterol, %	
Borderline high cholesterol, %	
Diabetes, %	
Prediabetes, %	
Psychological distress, %	NHIS 2009–2010, 2011–2012, 2013–2014, 2015–2016
No. of deaths from CVD events	CDC WONDER 2009–2016
Post-CVD prevalence, %	NHIS 2006–2016
Post-CVD adults with CVD disability, %	NHIS 2006–2015
No. of smoking-related non-CVD deaths	CDC WONDER 1999–2016, SAMMEC
No. of diabetes-related non-CVD deaths	CDC WONDER 2008–2016
No. of distress-related non-CVD deaths	
No. of smoking-related non-CVD hospitalizations	HCUP: NHCS (2013–2014), NIS (2010–2015)
No. of diabetes-related non-CVD hospitalizations	
No. of distress-related non-CVD hospitalizations	
Non-CVD adults with hypertension-related disability, %	MEPS 2006–2015, plus SAMMEC for smoking-related disability
Non-CVD adults with smoking-related disability, %	
Non-CVD adults with diabetes-related disability, %	
Non-CVD adults with obesity-related disability, %	

Abbreviations: AHA, American Heart Association; CDC, Centers for Disease Control and Prevention; CHD, congestive heart disease; CVD, cardiovascular disease; MEPS, Medical Expenditure Panel Survey; HCUP, Healthcare Cost and Utilization Project; NHANES, National Health and Nutrition Examination Survey; NHIS, National Health Interview Survey; NIS, Nationwide Inpatient Sample; PRISM, Prevention Impacts Simulation Model; SAMMEC, Smoking-Attributable Mortality, Morbidity, and Economic Costs; WONDER, Wide-Ranging Online Data for Epidemiologic Research.

## Results

### Face validation

SMEs generally agreed with the model structure, parameter values, and data sources. SME review resulted in improvements in some documentation, and additional information was added to documentation about how parameter values were determined when there were gaps in available literature. Some parameters were updated based on SME input. Table 2 presents a summary of comments from SMEs and updates made to the model based on those comments. For example, because trans fats have largely been eliminated from the US food supply, these model parameters were updated. Additionally, SMEs shared more recent peer-reviewed published estimates of the effect of controlling prediabetes on diabetes onset. We therefore included these newer effect estimates in PRISM. We made these changes before conducting additional types of validation. In the second round of review as part of the CDC clearance process, we made the following model updates based on CDC SME comments: created separate levers for quality acute care and quality rehabilitation care, which had previously been combined; removed the use of aspirin for primary prevention of CVD based on updated evidence; and updated sources for physical activity in schools and child care levers.

**Table 2:** Summary of Face Validation Comments From SME Reviews and Updates to the Model

SME Subject Area	Summary of Comments	Updates to the Model Based on Comments
CVD	Newer meta-analyses could be used to update parameter for the relative risk of CVD from aspirin use.	Updated the model parameter for relative risk of CVD from aspirin use.
Nutrition, physical activity, and obesity	• Two parameters were based on SME opinion that could be updated based on the literature. However, the literature may not have the relevant estimates, in which case, better describing the process used by SMEs to estimate the parameter is important. • Trans fats have largely been taken out of the US food supply over the past 5 years, so updating this trend in the model is important.	• Updated the parameters for relative risk of CVD from low fruit and vegetable consumption and relative risk of CVD from trans-fat fraction of diet based on new references. • Updated the fraction of trans fat in the diet to reflect new trends.
Smoking	• For parameters provided by past SMEs, how SMEs derived estimates is unclear. Ideally, parameters should be grounded in the literature and, where that is not feasible, as much detail as possible should be included on the process SMEs used to estimate the parameter. • More clarity is needed on the definitions of levers and parameters, such as what type of smoking ban is specified and whether the model includes all tobacco products or only cigarettes. • Some upper and lower bounds may be inappropriately high or low. Most notably, certain interventions may not plausibly create worse health outcomes (eg, the impact of smoke-free workplaces).	• Updated PRISM documentation for all parameters based on expert opinion to clarify how experts determined each parameter. • Added more detailed definitions of each PRISM lever to the PRISM reference material. • Updated the upper and lower bounds of the sensitivity analysis for each parameter and revised bounds to constrain interventions to producing no change or better outcomes.
Diabetes	The estimate for the impact of controlling prediabetes on diabetes onset may be too low.	Updated the parameter for the impact of controlling prediabetes on diabetes based on new references.

Abbreviations: CVD, cardiovascular disease; PRISM, Prevention Impacts Simulation Model; SME, subject matter expert.

### Internal validation

A coding expert who did not create the model code reviewed all model code and equations for consistency and accuracy. She identified 20 potential issues that required discussion with the model programmer. After discussion, all 20 were determined to not be issues and correctly programmed.

**Figure 2:**
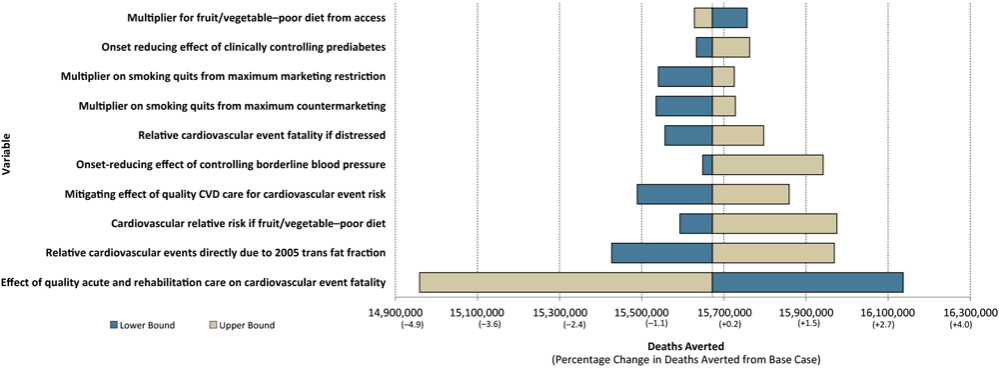
Results of 1-way sensitivity analysis of PRISM parameters that affected the impact of PRISM strategies on preventing cardiovascular deaths by more than ±0.5% compared with the estimated deaths averted from the base run (n = 15,672,020). Abbreviation: CVD, cardiovascular disease. (Minimum and maximum values used in 1-way sensitivity analysis are available upon request.)

Of the 86 parameters tested in 1-way sensitivity analyses, we found 10 PRISM parameters that, when varied across the full range of plausible values, affected the estimated impact of PRISM strategies on CVD deaths by 0.5% or more (Figure 2). (Parameter values and their outcomes and results from the other 76 parameters are available upon request.) The simulated number of cumulative deaths averted by moving all PRISM levers was 15.67 million for the period 2018 through 2040. CVD deaths were most sensitive to varying the effect of high-quality acute and rehabilitation CVD care; the upper bound (0.90) for this parameter resulted in a reduction in CVD deaths of 4.6%, while the lower bound (0.43) resulted in an increase in CVD deaths of approximately 3.0%. For all other inputs, the estimated number of deaths varied by less than 2% above or below the base estimate of 15.67 million. This variation is in contrast to other models examined in cross validation, which had parameters that affected results by 40% to 100% in sensitivity analysis (12).

**Table:** 

Analysis	Cumulative CVD Deaths Averted (2018–2040)	
	Lower Bound	Upper Bound
Multiplier for fruit/vegetable–poor diet from access	15,592,960	15,975,310
Onset-reducing effect of clinically controlling prediabetes	15,633,170	15,763,020
Multiplier on smoking quits from maximum marketing restriction	15,540,520	15,725,640
Multiplier on smoking quits from maximum countermarketing	15,540,520	15,725,640
Relative cardiovascular event fatality if distressed	15,556,430	15,797,190
Onset-reducing effect of controlling borderline blood pressure	15,648,570	15,941,810
Mitigating effect of quality CVD care for cardiovascular event risk	15,489,010	15,859,030
Cardiovascular relative risk if fruit/vegetable–poor diet	15,592,960	15,975,310
Relative cardiovascular events directly due to 2005 trans fat fraction	15,426,820	15,969,070
Effect of quality acute and rehabilitation care on cardiovascular event fatality	14,959,130	16,136,860

### Cross validation

Cross validation of PRISM version 3q with the CVD Policy Model and ModelHealth showed a high degree of consistency across models in the estimated reduction in cardiovascular events and cardiovascular mortality for achieving 5-year Million Hearts goals compared with the number of events and mortality simulated in each model (Table 3). However, PRISM showed a larger impact of increasing blood pressure control on reducing deaths than did the other models.

**Table 3:** Simulated Change in Number of CVD Events and Deaths for Achieving 5-Year Million Hearts Goals: Outcomes From 3 CVD Models for US Adult Population

Goal	CVD Events (% Change from Base Run)			CVD Deaths (% Change from Base Run)		
	PRISM	CVDPM	MH	PRISM	CVDPM	MH
Aspirin use for secondary CVD prevention	−0.17	−0.28	−0.21	−0.18	−0.16	−0.22
Blood pressure control	−2.85	−1.98	−2.69	−5.19	−1.51	−2.24
Cholesterol management	−0.83	−0.82	−0.89	−1.32	−0.49	−0.96
Mean daily sodium intake reduction	−1.04	−1.81	−2.91	−1.87	−1.34	−1.32

Notes: CVD, cardiovascular disease; CVDPM, CVD Policy Model; MH, ModelHealth; PRISM, Prevention Impacts Simulation Model.

Table 4 shows a comparison of results between PRISM and individual studies. For universal tobacco counseling, estimated reductions in smoking prevalence between 2015 and 2040 from PRISM were similar to results from Maciosek et al (11), but cost estimates differed substantially. PRISM interventions cost estimates were twice as high as those reported by Maciosek et al (11), whereas the simulated reduction in medical costs from PRISM were substantially lower ($166 million vs $2.7 billion). In fact, although Maciosek et al found cost savings, the PRISM analysis showed a net increase in costs of $708 million, or about $140,000 per death averted.

**Table 4:** Cross-Validation Analyses Comparing PRISM to Published Estimates

Outcome	Study Estimate	PRISM Estimate
**Tobacco counseling intervention for US adult smokers (Maciosek et al, 2017 [** 11 **])^a^^,^ b**		
Percentage change in smoking prevalence	−3.8	−4.7
Counseling and cessation medication costs, 2012 dollars	427 million	874 million
Change in medical costs, 2012 dollars	−2.7 billion	−166 million
Change in total costs, 2012 dollars	−2.3 billion	708 million
Premature deaths prevented	69,901	5,074
**Sugar-sweetened beverage tax applied for US adults (Wang et al, 2012 [** 12 **])^c^^,^ d**		
Percentage change in diabetes incidence/prevalence	−2.6	−0.3
Strokes prevented	8,000	4,463
Change in total costs, 2010 dollars	−17.1 billion	−893 million
Premature deaths prevented	26,000	4,000
**Physical activity in early childhood education for United States (Gortmaker et al, 2015 [** 13 **])^e^^,^ f**		
First-year implementation cost, 2014 dollars	4.8 million	2.1 million
Change in medical costs, 2014 dollars	−51.6 million	−6.1 million
Change in net costs, 2014 dollars	−43.2 million	13.9 million
**Active physical education in schools for United States (Gortmaker et al, 2015 [** 13 **])^g^^,^ h**		
First-year implementation cost, 2014 dollars	70.7 million	57.1 million
Change in medical costs, 2014 dollars	−60.5 million	−39.1 million
Change in net costs, 2014 dollars	175 million	499 million

Abbreviation: PRISM, Prevention Impacts Simulation Model.

a Costs are the current value of costs in 2012 dollars, using a 3% annual discount rate. Estimates from Maciosek et al follow an adult cohort for their lifetime; PRISM estimates are for providing smoking quit services to all adult smokers for a 30-year period.

b Results are for adult counseling vs no adult counseling (11). In contrast, PRISM estimates compare increased use of smoking quit services with current rates of use for the analysis horizon.

c Wang et al (12) reported estimated effects of a penny-per-ounce tax on sugar-sweetened beverages on diabetes incidence rates and stroke incidence for US adults aged 25 to 64 years over a 10-year period. Authors used the Coronary Heart Disease Policy Model to estimate the impact of simulated changes in diabetes and body mass index on cardiovascular disease, costs, and deaths for 2010 through 2020. Cost year is not reported, but we assumed published costs were in 2010 dollars. Both Wang et al and PRISM costs were discounted using a 3% annual discount rate.

d PRISM analysis assumes an increase of 1.9% in the energy-dense food pricing lever, estimated as the percentage change in the price from the tax (estimated as 7.8% by Silver et al 2017 [14]) scaled by the fraction of energy-dense food consumption made up by sugar-sweetened beverages (estimated as 24.5% by Huth et al 2013 [15]). PRISM cost estimates reflect the present value of medical costs in 2010 dollars, with future costs discounted using a 3% annual discount rate.

e Gortmaker et al (13) reported estimates of the impact of requiring public elementary schools to devote ≥50% of physical education class time to moderate and vigorous physical activity. Costs were reported in 2014 dollars. The authors did not report discounting of costs.

f PRISM analysis assumes a 0.53 lever movement of the physical activity in schools lever based on a highintensity intervention that applies to the proportion of children in elementary school. Costs were reported in 2014 dollars and were discounted by 3% annually.

g Gortmaker et al (13) reported estimates of the impact of requiring early child care centers to provide 90 minutes of moderate to vigorous physical activity over the course of the program day. Costs were reported in 2014 dollars. The authors did not report discounting of costs.

h PRISM analysis assumes a 0.70 lever movement of the physical activity in child care lever based on a high-intensity intervention that applies to all children in child care. Costs were reported in 2014 dollars and were discounted by 3% annually.

Table 4 also shows results from Wang et al (12) compared with results from the PRISM simulation of a penny-per-ounce tax on sugar sweetened beverages. PRISM estimated a smaller impact on diabetes prevalence and strokes prevented and a substantially lower impact on medical costs saved ($893 million savings vs $17.1 billion savings) and premature deaths prevented (4,000 vs 26,000).

Table 4 presents results from Gortmaker et al’s (13) simulation of an intervention for physical activity in early childhood education compared with results from moving the PRISM physical activity in childcare lever to its maximum. PRISM estimated lower first-year implementation costs, but substantially smaller medical cost savings over 10 years ($6.1 million vs $51.6 million) than Gortmaker et al (13). Results from the study by Gortmaker et al (13) of an intervention targeting active physical education in schools were compared with those from PRISM, and findings again showed that PRISM estimated lower first-year implementation costs. Estimated medical cost savings over 10 years were smaller in PRISM ($39.1 million vs $60.5 million), and total net costs inclusive of implementation costs were much higher in PRISM ($499 million vs $175 million).

Findings from all cross-validation exercises suggest that PRISM produces results that are closely aligned with the CVD event and death estimates for Million Hearts interventions from the CVD Policy Model and ModelHealth. However, for tobacco counseling, sugar-sweetened beverage tax, and childhood physical activity interventions, PRISM produced substantially lower estimates of the impact on cost savings than other published models, which estimated much higher cost savings from interventions.

### External validation

We analyzed how PRISM version 3q1a output compared with national surveillance data for selected years between 1996 and 2016, where data were selected based on availability, across 25 risk factors and outcomes (Table 1). PRISM estimates mostly mapped well to 21 of the 25 variables. Four variables showed trends in surveillance data that deviated from PRISM projections: obesity prevalence in adults, deaths from CVD events, diabetes-related non-CVD deaths, and psychological distress-related non-CVD deaths. To update obesity prevalence in adults, deaths from CVD events, diabetes-related non-CVD deaths, and psychological distress-related non-CVD deaths, we recalibrated multipliers in PRISM to better capture the trends observed in surveillance data. This re-calibration changed the underlying trends of these variables and impacts their analysis in the model. After recalibration, PRISM estimates matched surveillance data with no effect on the fit of other variables to surveillance data. We considered 3 additional output measures for recalibration: secondhand smoke exposure, borderline high cholesterol prevalence, and smoking-related non-CVD hospitalization. However, the study team determined that although these measures exhibited variation that differed from PRISM estimates, they were not consistent substantial divergences in trends from PRISM estimates. Therefore, in the interest of not overcalibrating the model, these outcomes were not recalibrated in the model.

## Discussion

Simulation models can provide useful insights for guiding health policy planning and evaluation decisions. However, the usefulness of models for planning and evaluation rests on whether a potential user has confidence and trust in the model’s predictions. Therefore, we assessed the face, internal, cross, and external validity of PRISM. This work resulted in some refinements in the model and may also be useful for guiding decisions about whether to use PRISM for community-level strategic planning, policy analysis, or evaluation.

Face validation findings confirmed that PRISM equations and parameters are based on the latest scientific knowledge. Internal validation findings showed that uncertainty in the model parameter values, taken one at a time, have little impact on the model’s estimated cumulative number of deaths for 2018 through 2040 compared with the base run estimate. The estimated impact of strategies in the model was most sensitive to the values used for the effect of quality acute and rehabilitation care on CVD event fatality; across the plausible range of values for this input, the cumulative estimated number of deaths averted by PRISM strategies varied from 14.95 million to 16.15 million. For all other inputs, the estimated number of deaths varied by less than 2% above or below the base run cumulative number of deaths, 15.67 million. Thus, sensitivity analysis results show that PRISM outcomes are minimally influenced by the values of any single parameter in the model, suggesting that PRISM is a relatively stable CVD model. However, because the base case is so large (the estimated impact of all PRISM strategies), a small percentage still means a large number of deaths. Findings suggest that PRISM results are quite robust to uncertainty in input values used in the model. This finding was not surprising because PRISM models a large system of risk factors, and no individual factor substantially influences the system. These findings can assure users that PRISM estimates are not considerably impacted by uncertainty in any one of the parameters.

Cross validation suggested that PRISM may produce more conservative (ie, lower) impact estimates than comparable models, largely because of differences in cost modeling methods and assumptions. For example, PRISM includes costs of interventions to increase the use of smoking quit services, whereas Maciosek et al (11) did not. Additionally, in PRISM, medical cost savings are estimated for smoking-attributable events using an attributable-fraction costing approach, while Maciosek et al estimates savings in any medical spending attributable to current and former smoking. Because of these methodologic differences, Maciosek et al estimated cost savings that are higher than those calculated using PRISM’s attributable fraction-costing approach (3). PRISM also uses more conservative estimates of the impact of interventions. For example, Wang et al (12) used substantially higher estimates of the impact of a tax on diabetes incidence. They assumed that sugar-sweetened beverage consumption directly increases diabetes incidence and also raises incidence through higher calorie consumption. In sensitivity analysis, Wang et al estimated that even if all calories from sugar-sweetened beverages were replaced with other caloric beverages, the tax would still lead to a roughly $6 million reduction in medical costs through the direct impact on diabetes. PRISM assumes a more conservative impact of energy-dense foods consumption on diabetes incidence. We do not know whether these differences reflect overestimates of the cost and health benefits of interventions in some models or underestimates in others, including PRISM. Additional refinements of parameter estimates and triangulation between models using various methods would help to narrow relevant discrepancies between various approaches.

External validation showed that PRISM projections aligned closely with surveillance data and trends for most risk factors and outcomes. Additionally, for the 4 variables with trends that deviated from PRISM estimates, we recalibrated the model to better match surveillance data. The concordance of PRISM output and national surveillance measurements and trends indicates that PRISM models chronic disease trends well through 2016 and beyond.

This comprehensive validation of PRISM demonstrates that the model is based on the most up-to-date science, is not excessively sensitive to uncertainty in parameter values, produces conservative estimates of health and economic impacts, and maps well with trends in risk factors and outcomes. These results should assure health policy leaders that PRISM can be a useful tool to support their planning and evaluation efforts. Compared with other policy models, PRISM includes a broader range of risk factors for CVD, including tobacco use, nutrition, physical activity, distress, and air pollution. Therefore, it may be useful to examine results from multiple models to understand the range of possible estimates under less conservative assumptions. PRISM also allows users to consider the health, mortality, and economic impacts of implementing up to 32 intervention strategies. However, there are aspects of population heath that PRISM does not model because of limitations in data or model structure. Most notably, PRISM cannot be used to model health disparities and does not model infectious disease such as COVID-19. Although no model can perfectly account for unexpected infectious disease events such as the COVID-19 pandemic, PRISM has the flexibility to allow the user to update baseline levels of relevant inputs such as control of chronic conditions or diet in response to these changes. Future updates to PRISM should include population measures that allow for analysis of health disparities. PRISM has undergone rigorous validation and is now openly accessible in a user-friendly online interface for health policy leaders to use.
